# Clinical Outcomes of COPD Patients Hospitalized for SARS-Cov-2 Infection During the Omicron Era: Comparative Effectiveness of Initiating Remdesivir in Addition to Corticosteroids Versus Corticosteroids Alone

**DOI:** 10.3390/v17111438

**Published:** 2025-10-29

**Authors:** Neera Ahuja, Heng Jiang, Marc Milano, Roman Casciano, Ananth Kadambi, Thomas Oppelt, Fariborz Rezai, Martin Kolditz, Veronika Müller, Essy Mozaffari

**Affiliations:** 1 Department of Internal Medicine, School of Medicine, Stanford University, Palo Alto, CA 94305, USA; 2 Evidence and Access, Certara, 75008 Paris, France; 3 Newark Beth Israel Medical Center, Somerset, Somerville, NJ 08876, USA; 4 Evidence and Access, Certara, Radnor, PA 19087, USA; 5 Gilead Sciences Inc., Foster City, CA 94404, USA; thomas.oppelt@gilead.com; 6 Rutgers Medical School, Critical Care Medicine, RWJ Barnabas Health, West Orange, NJ 07052, USA; 7 Medical Department I, University Hospital Carl Gustav Carus of TU Dresden, 01307 Dresden, Germany; 8 Department of Pulmonology, Semmelweis University, 1083 Budapest, Hungary; 9 Medical Affairs, Gilead Sciences, Foster City, CA 94404, USA

**Keywords:** COPD, corticosteroids, COVID-19, remdesivir, SARS-CoV-2, mortality, real-world evidence

## Abstract

Patients with chronic obstructive pulmonary disease (COPD) are vulnerable to developing severe SARS-CoV-2 infection. This retrospective study evaluated the effectiveness of remdesivir (RDV) initiated with corticosteroids (CCS) versus CCS only in patients with COPD hospitalized for SARS-CoV-2 infection during the Omicron period from December 2021 to February 2024. The analysis used patient-level data from the large, geographically diverse, US hospital administrative billing PINC AI healthcare database. Inverse probability of treatment weighting was used to adjust for potential confounding and enable a scientifically robust comparative assessment of differences in outcomes between treatment groups. Initiation of RDV with CCS upon admission for SARS-CoV-2 infection was associated with a lower mortality risk at 14 and 28 days with an overall adjusted hazard ratio [95% CI] of 0.74 [0.68–0.80] and 0.76 [0.71–0.82], respectively, compared to initiation of CCS only. The combination of RDV and CCS was also associated with a lower mortality risk at 14 and 28 days for patients across baseline oxygen requirements compared to CCS only. These results highlight the benefit of timely RDV treatment in COPD patients hospitalized with SARS-CoV-2 infection and underscore the value of considering established treatment paradigms in the context of the most recent collective evidence.

## 1. Introduction

Patients with chronic obstructive pulmonary disease (COPD) have a higher risk of SARS-CoV-2 infection, a worse short-term prognosis for SARS-CoV-2 outcomes compared to those without COPD, and an increased risk of long-term mortality due to SARS-CoV-2 [1,2]. In addition, patients with COPD may have a dysregulated innate immune response to viral infections [3].

In 2020, the RECOVERY study recommended the use of the corticosteroid dexamethasone (DEX) in patients hospitalized with COVID-19 receiving invasive mechanical ventilation (IMV) or oxygen alone at randomization, but not among those receiving no respiratory support [4]. The American College of Chest Physicians pointed to the RECOVERY study in their Guidelines and Research Summaries in 2020 stating that “dexamethasone is the first therapeutic agent shown to reduce mortality due to COVID-19” [5]. In 2021 the European Respiratory Society recommended systemic corticosteroids (CCS) in patients hospitalized for COVID-19 requiring supplementary oxygen or ventilatory support [6]. Additionally, if underlying COPD is exacerbated in the context of an acute respiratory tract infection, a short course of CCS is considered standard of care [7,8].

Early treatment with the antiviral remdesivir (RDV) reduced mortality in patients hospitalized for SARS-CoV-2 infection, regardless of their supplemental oxygen requirements [9]. Initiating RDV and DEX upon admission in patients hospitalized for SARS-CoV-2 infection resulted in a significant reduction in 14- and 28-day mortality compared to those initiated on DEX only [10]. The evidence from infectious disease studies, in which the survival benefit of CCS in cases of severe infection manifests only when combined with antimicrobials, is consistent with this benefit of RDV in SARS-CoV-2 infection [11,12,13]. Prior research also showed a significant reduction in 30-day COVID-19-related and all-cause readmission for RDV-treated patients hospitalized for COVID-19, suggesting that the benefit of RDV treatment in these patients may extend beyond hospitalization [9]. In a recently published systemic literature review, the totality-of-evidence from randomized clinical trials and real-world studies indicated that treatment with RDV provides a significant survival benefit in patients hospitalized with SARS-CoV-2 infection regardless of disease severity or oxygen support requirements at admission [9]. The systemic literature review also demonstrated that RDV treatment shortens time to clinical improvement and significantly lowers the risk of hospital readmission [9].

Given the efficacy and effectiveness of RDV in treating SARS-CoV-2 infection, our objective was to assess the clinical benefit of RDV in patients with COPD who are at high risk of severe COVID-19 due to their underlying condition. Specifically, we conducted a comparative effectiveness study using real-world data to evaluate the clinical outcomes in patients with COPD hospitalized for SARS-CoV-2 infection who either initiated RDV with CCS upon admission or were treated with CCS alone.

## 2. Materials and Methods

### 2.1. Study Design and Data Source

This retrospective, comparative effectiveness cohort study used patient-level data from the large, geographically diverse, Health Insurance Portability and Accountability Act-compliant, all-payer hospital administrative billing PINC AI healthcare database (www.pinc-ai.com, accessed on 1 February 2025) [14]. The PINC AI database covers 25% of hospitalizations from 48 US states and includes diagnoses at hospital admission and discharge, and demographics and patient-level information for each day of the hospital stay. All baseline variables were examined in the first two days of hospitalization. This study was conducted with patient information from December 2021 to February 2024 during the Omicron period.

### 2.2. Study Population

**Figure 1:**
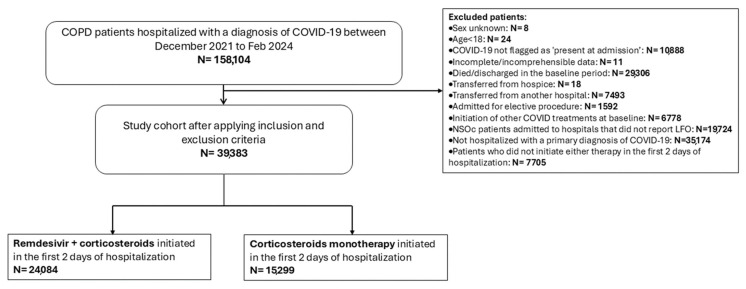
Study Population. COPD, chronic obstructive pulmonary disease; COVID-19, coronavirus disease 2019; LFO, low flow oxygen; NSOc, no supplemental oxygen charges.

The study population included adult patients ≥18 years of age with COPD hospitalized for COVID-19 (International Classification of Diseases, 10th Revision, Clinical Modification [ICD-10-CM] code U07.1) as a primary discharge diagnosis that was also flagged as “present on admission” (Figure 1). All patients received at least one dose of either the combination of RDV and CCS or CCS only (prednisone, prednisolone, methylprednisolone, hydrocortisone, or DEX) during the baseline period, defined as the first two days of their hospitalization. Patients with COPD were identified by the ICD-10 diagnosis codes J43 and J44. Other key study variables extracted from the database are defined in Table S1. Patients were stratified according to baseline supplemental oxygen requirements including no supplemental oxygen charges (NSOc), low-flow oxygen (LFO), high-flow oxygen/non-invasive mechanical ventilation (HFO/NIV), and IMV/extracorporeal membrane oxygenation (ECMO). Patients who did not require supplemental oxygen (NSOc) were identified using methods previously reported [10]. Stratifying the patients by their baseline supplemental oxygen requirements supported inclusion of patients across the spectrum of COVID-19 severity.

Several exclusion criteria were implemented in this evaluation. To avoid compromising the definition of NSOc patients, we excluded patients admitted to hospitals which did not explicitly report charges for supplemental oxygen. Furthermore, we excluded patients who were not hospitalized due to a primary diagnosis of COVID-19, or who were discharged or died during the baseline period [15]. Other exclusion criteria included incomplete/erroneous data, transfer from another hospital or hospice, admission for elective procedures, and initiation of other treatments for SARS-CoV-2 infection during the baseline period including baricitinib or tocilizumab. Exclusion of patients receiving antivirals other than RDV was done in order to focus this study specifically on the change in mortality risk in patients receiving RDV. For patients who had multiple hospitalizations for SARS-CoV-2 infection, only the first hospitalization during the study period was considered. Finally, to ensure that all patients discharged alive would have at least 30 days of follow-up post-discharge, we excluded patients who were discharged less than 30 days after the end of the study period (February 2024).

### 2.3. Study Outcomes and Variables

The primary outcomes for this study were 14- and 28-day all-cause in-hospital mortality (defined as a discharge status of “expired” or “hospice”). Baseline supplemental oxygen requirements were characterized as NSOc, LFO, HFO/NIV, or IMV/ECMO. The follow-up period started the day after baseline until day 28 or a discharge status of expired or hospice, transfer to another hospital, or addition of RDV after the first two days of hospitalization in the CCS only group, whichever came first. All-cause inpatient mortality was assessed at 14 and 28 days after the baseline period.

Baseline covariates captured in this study included demographics (age, sex, race, and ethnicity), key comorbidities (obesity, cardiovascular disease, diabetes, renal disease, immunocompromised condition, cancer, and lung cancer), hospital characteristics (bed size, location and region, and ward upon admission), and baseline supplemental oxygen requirements. These covariates are clinically relevant to SARS-CoV-2-related outcomes by assessing differences in demographics, severity of disease, and variations in hospital care.

### 2.4. Statistical Analysis

Analyses were conducted for the overall population and stratified by baseline supplemental oxygen requirements for each of the two treatment groups which included those who received RDV with CCS or CCS only upon admission. The inverse probability of treatment weighting (IPTW) approach was used to adjust for potential confounding and allow for a scientifically robust comparative assessment of the differences in the outcomes between the two treatment groups [16].

The IPTW weights were derived from the propensity scores (PS), which represent the probability of receiving the target treatment. In this study, PS was estimated using separate logistic regression models for each category of baseline oxygen support requirement, with RDV in addition to CCS exposure as the dependent variable. Each model incorporated baseline covariates specific to the respective treatment groups (Table 1). This stratified modeling approach was employed to ensure comparability within each oxygen support category. IPTW was then used to balance the RDV + CCS and CCS only treatment groups. Extreme PS values (<0.05 or >0.95) were excluded. Patient characteristics at index hospitalization were balanced post-IPTW with an absolute standardized mean difference <0.15 between the two groups (Table 1).

A Cox proportional hazards model was used to evaluate time to 14- and 28-day in-hospital all-cause mortality, and adjusted hazard ratios (aHRs) with 95% confidence intervals (CIs) were reported. The models accounted for hospital-level clustering effects using a robust sandwich variance estimator and adjusted for key covariates, including age, admission month, hospital admission ward (documented bed charges for intensive care unit/step-down unit versus general ward), and time-varying covariates for treatments initiated after the baseline period, such as baricitinib, tocilizumab, oral antivirals, or CCS other than DEX. Admission month was included as a key covariate so that patients hospitalized for SARs-CoV-2 infection in the early Omicron period were not compared to those from the late Omicron period. Further, a sensitivity analysis excluding those in the 18–49 year age group was performed.

All statistical analyses were conducted using SAS Version 9.4 (SAS Institute Inc., Cary, NC, USA). All statistical tests were two-sided, and the level of significance was *p* ≤ 0.05 without adjustment for multiple testing.

## 3. Results

During the study period, 158,104 patients with COPD were hospitalized for SARS-CoV-2 infection in 1000 hospitals. Of these, 39,383 met the inclusion criteria for this study (Figure 1). Of the included patients, 24,084 (61%) were treated with both RDV + CCS and 15,299 (39%) were treated with CCS only during the baseline period.

The baseline demographics and hospital characteristics of the study population before and after IPTW are shown in Table 1. Before IPTW, patients in the group treated with RDV + CCS and the CCS only group, respectively, were predominantly ≥65 years of age (77.6%, 78.6%), female (54.3%, 54.4%), White (82.0%, 81.1%), and non-Hispanic (88.2%, 88.3%), and 19.7%, 19.5%, respectively, had immunocompromising conditions. Before IPTW, patients in the group treated with RDV + CCS were less likely to have renal disease compared to the CCS only group (26.6% vs. 36.0%), which is to be expected given the RDV contraindication in patients with renal disease, and had a lower mean Charlson comorbidity index (3.5 vs. 3.9). Before IPTW, most patients received LFO (39%), followed by HFO/NIV (23%), and IMV/ECMO (2.3%).

After IPTW, characteristics were well-balanced. Most patients were ≥65 years of age (78%), female (54%), White (82%), and non-Hispanic (88%). After IPTW, proportions were similar, with most patients receiving LFO (39%), followed by HFO/NIV (25%), and IMV/ECMO (3.4%). The absolute standardized mean difference in renal disease for the CCS only group vs. the RDV + CCS group after IPTW was <0.10, indicating that the two groups were well-balanced with respect to this variable.

**Figure 2:**
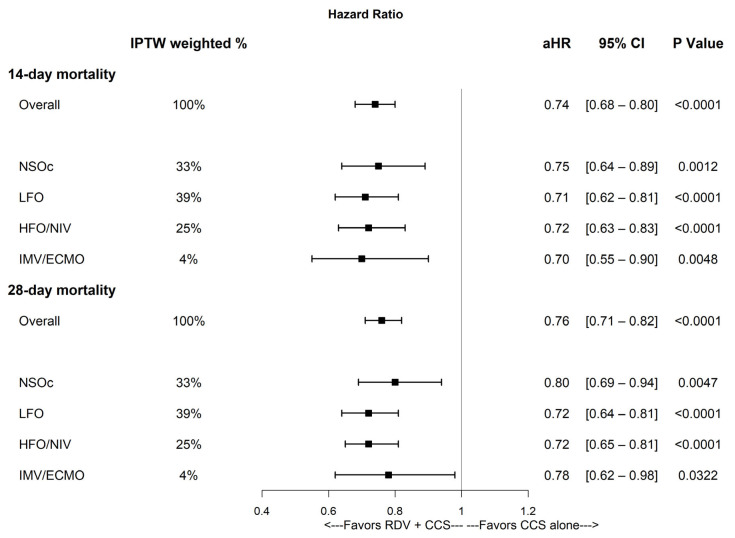
14- and 28-day mortality in patients with COPD hospitalized with SARS-CoV-2 infection initiated on both RDV + CCS or CCS alone upon admission, stratified by supplemental oxygen requirements (after IPTW). Estimates adjusted for age, admission month, hospital admission ward (documented bed charges for ICU/step-down unit versus general ward), and time-varying covariates for treatments initiated after the baseline period, such as baricitinib, tocilizumab, oral antivirals, or CCS other than DEX. aHR, adjusted hazard ratio; CCS, corticosteroids; CI, confidence interval; HFO/NIV, high flow oxygen/non-invasive ventilation; IMV/ECMO, invasive mechanical ventilation/extracorporeal membrane oxygenation; IPTW, inverse probability of treatment weighting; LFO, low flow oxygen; NSOc, no supplemental oxygen charges; RDV, remdesivir.

Initiation of both RDV + CCS upon admission in patients with COPD hospitalized for SARS-CoV-2 infection was associated with a lower mortality risk at 14 and 28 days with an overall aHR [95% CI] of 0.74 [0.68–0.80] and 0.76 [0.71–0.82], respectively, compared to those receiving CCS only (Figure 2). Treatment initiation with RDV was also associated with a lower mortality risk at 14 days (aHR [95% CI]) for patients not receiving supplemental oxygen, (NSOc), (0.75 [0.64–0.89]), LFO (0.71 [0.62–0.81]), HFO/NIV (0.72 [0.63–0.83]), and IMV/ECMO (0.70 [0.55–0.90]), compared to those receiving CCS only; and at 28 days for patients receiving NSOc (0.80 [0.69–0.94]), LFO (0.72 [0.64–0.81]), HFO/NIV (0.72 [0.65–0.81]), and IMV/ECMO (0.78 [0.62–0.98]), compared to those receiving CCS only.

We also did an evaluation to ascertain whether patients in the 18–49 year age group were primarily suffering from emphysema with severe alpha-1 antitrypsin deficiency, by examining the reported diagnosis codes for these patients. We identified that of the 824 patients in this age group (313 CCS only, 511 RDV + CCS), 95 patients (42 CCS only, 53 RDV + CCS) representing 0.2% of the full study cohort and 11.5% of the 18–49 year age group, reported a diagnosis of emphysema; while the vast majority of patients reported a diagnosis of COPD. As such, the impact of including these patients is expected to be minimal. We then conducted a sensitivity analysis to confirm this, by excluding all patients in the 18–49 year age group, and findings were unchanged (Table S2).

## 4. Discussion

Although the COVID-19 pandemic era is behind us, SARS-CoV-2 remains prevalent and continues to burden health systems worldwide, especially in vulnerable individuals who are immunocompromised, elderly, or have comorbidities such as COPD. Early in the pandemic, treatment guidelines for SARS-CoV-2 infection were largely based on the initial randomized clinical trials (RCTs). Given that enrollment into RCT’s became difficult during the latter part of the pandemic, treatment guidelines were challenging to update. Hence, retrospective studies have been key to providing clinical insights for the management of patients hospitalized for SARS-CoV-2 infection. Consequently, this has led to more evidence generation around the effectiveness of antivirals based on observational research, and also in specific vulnerable populations not studied in the earlier RCTs. For example, a recent comprehensive review summarized the totality of the evidence for RDV, including both RCTs and observational studies, depicting the effectiveness of RDV across several key clinical outcomes [9]. In addition, a recent study identified the main risk factor for COVID-19 disease progression as not having received antivirals or monoclonal antibodies [17]. Since we have been managing and will continue to manage vulnerable populations hospitalized for SARS-CoV-2 infection, it is critical to evaluate the effectiveness of therapeutic approaches in real-time, considering the evolution of numerous variants and sub-variants of the virus over the past five years. To address this need for ongoing evidence generation, we conducted an analysis of real-world data to provide insights into the effectiveness of RDV treatment in patients with COPD hospitalized for SARS-CoV-2 infection in the current era.

This study builds on a previous report that showed a lower mortality risk for patients hospitalized with SARS-CoV-2 infection who were treated with RDV + DEX versus DEX alone [10]. The present study is focused on vulnerable patients with COPD, treatment with other CCS in addition to DEX, and includes more recent data through February 2024. Using IPTW, a standard method in comparative effectiveness research [16], we observed a statistically significant association in lowering mortality risk when patients with COPD hospitalized with SARS-CoV-2 infection were initiated on RDV upon admission across all supplemental oxygen requirements (Figure 2). The overall 28-day mortality HR was 0.76, corresponding to a reduction in mortality risk of 24% in patients initiated on both RDV and CCS compared to patients receiving CCS only. The 28-day mortality risk is also reduced by 20%, 28%, 28%, and 22%, in patients receiving NSOc, LFO, HFO/NIV, and IMV/ECMO, respectively. These results are consistent with prior reports of mortality rate reduction associated with RDV treatment in vulnerable populations hospitalized with SARS-CoV-2 infection [9]. Furthermore, these results are consistent with prior evidence indicating that the survival benefit of CCS manifests only when combined with antivirals [11,12,13].

Patients with COPD have a higher risk for severe COVID-19 outcomes [1,2] and our study highlights the benefits of targeting the SARS-CoV-2 virus directly, with RDV, in this vulnerable patient population. Management of acute COPD exacerbations that can occur during SARS-CoV-2 infection in patients with COPD, is critical to quality of life and COPD progression [18,19]. It is notable that COPD acute exacerbations are managed with inhaled or systemic CCS, but CCS can also cause adverse effects that may be related to cumulative dose [19,20].

The reduction in mortality risk in IMV/ECMO patients with SARS-CoV-2 infection who were initiated on both RDV + CCS is noteworthy due to a prior lack of consensus on treatment in this subpopulation. In their 2023 guidelines, the World Health Organization, issued a “conditional recommendation” and “suggested treatment with remdesivir” in patients with severe COVID-19 [21]. In a 2022 German treatment guideline, the authors state that “The use of remdesivir in ventilated patients is not appropriate due to lack of any clinical benefit in this population” [22]. However, findings from prior real-world effectiveness studies and the results of this study provide evidence that RDV reduces mortality in IMV/ECMO patients [9,10] based on clinicians’ use of RDV in clinical practice. This result can potentially be explained by an antiviral’s ability to reduce the ongoing viral replication reported to occur in patients requiring mechanical ventilation [23]; however, information regarding ongoing viral replication was not available in the database. The consistency across real-world studies highlights their importance as a source to fill data gaps around real world effectiveness in infectious disease more broadly, e.g., in subpopulations of patients who were not enrolled in sufficient numbers to enable rigorous evaluation in RCTs and when conducting RCTs is not feasible.

Our findings also support timely initiation of RDV in patients with COPD hospitalized for SARS-CoV-2 infection who do not require supplemental oxygen (NSOc). The World Health Organization treatment guidelines provide a “conditional recommendation against” systemic corticosteroids for patients with non-severe COVID-19 [21] and other guidelines point to findings that DEX showed no beneficial effects in hospitalized patients with COVID-19 without any oxygen requirement [22]. Despite this evidence-based guideline, in this study, nearly 90% of COPD patients in the CCS-only group who did not require supplemental oxygen continued their CCS treatment regimen without the addition of RDV throughout their hospitalization. The RECOVERY study demonstrated no evidence of benefit in DEX-treated SARS-CoV-2 infected patients not requiring supplemental oxygen at randomization with a reported 28-day mortality in DEX-treated patients of 17.8% versus 14.0% in those who did not receive DEX [4]. It is possible that the antiviral activity of RDV in patients with no supplemental oxygen requirements and in patients with COPD improves viral clearance and attenuates any risk conferred by the appropriate use of CCS in these patients [10].

Our study has several key strengths. First, our evaluation is based on a large representative real-world population from a multi-hospital data source, as well as the application of IPTW methodology to balance inherently different groups and minimize confounding as much as possible [10]. The findings can be corroborated using additional real-world databases that include adequate granularity of data, sample size, and use of rigorous analytical methods to address potential biases associated with observational research.

Our study also has potential limitations typical of observational research and the majority of these have been previously described [10]. There remains the potential for residual confounding due to imbalances between treatment groups arising from unavailable data. For example, information about previous vaccination status, patient use of background medications prior to hospitalization (e.g., inhaled CCS), other potentially relevant lifestyle factors (e.g., cigarette smoking or socioeconomic factors), time to treatment from symptom onset, seronegative vs. seropositive status, or prior history of COVID-19 was unavailable in this database. The impact of these variables could not be accounted for which is a limitation of the study. To partially mitigate the potential confounding arising from the lack of information on whether the patients were already on any treatment at baseline, we incorporated prespecified baseline characteristics into the PS covariates (e.g., diagnoses, severity proxies, and concomitant inpatient treatments), so that IPTW assigned similar probabilities to patients with similar profiles. Absolute standardized mean difference for the covariates was <0.1, suggesting good post-weighting balance according to commonly accepted standards. As previously reported, these analyses were stratified by baseline supplemental oxygen requirements as a surrogate to ensure comparison across patients with similar SARS-CoV-2 infection severity [10]. However, in this study focused on the high-risk COPD patient population, it is important to point out that the database did not specify whether the oxygen requirements were based on COPD or SARS-CoV-2 infection severity. Therefore, this is a potential limitation of this study. Furthermore, with respect to vaccination status, as all patients included in this study were already hospitalized for COVID-19, reflecting failed protection from prior immunity, any biases introduced by inclusion of such patients in the analyses would be reduced. In addition, it could be expected that the PS matching approach which led to the balancing of the measured variables in this study (specifically age, and key comorbidities) is likely to have at least partially balanced out unmeasured variables such as vaccination and prior infection. Despite this, we acknowledge the study limitation that vaccination status may have impacted clinical decision-making as clinicians may have been more likely, for example, to prescribe RDV to those who were unvaccinated. This could have resulted in an underestimation of the benefit of RDV observed in this study, as unvaccinated patients would likely have worse outcomes unrelated to RDV administration. Additionally, we have no data on COPD severity and baseline COPD treatments in this population. Lastly, our analysis did not consider the impact of potential concomitant lung infection, although it should be noted that the impact is likely minimal, as such patients represented less than 0.1% of patients in both study groups of patients with COPD hospitalized for SARS-CoV-2 infection.

## 5. Conclusions

SARS-CoV-2 infection treatment guidelines for administration of RDV and/or CCS are largely based on RCTs performed during the pandemic era. Furthermore, guidelines either have no recommendation or a conditional recommendation for RDV in patients not requiring supplemental oxygen or those requiring IMV/ECMO. Analysis of real-world data from the pandemic era through the present provided foundational evidence to fill data gaps around the real-world effectiveness of treatments for SARS-CoV-2 infection and showed that RDV reduces mortality in patients with SARS-CoV-2 across all supplemental oxygen requirements. The current study demonstrated that RDV lowers mortality in patients with COPD by 20–28% across all supplemental oxygen requirements. The findings also revealed the use of CCS alone, without RDV, in nearly 90% of patients hospitalized with SARS-CoV-2 infection with no supplemental oxygen requirements, which is inconsistent with SARS-CoV-2 treatment guidelines. Collectively, these results suggest a continuing need to evolve and optimize SARS-CoV-2 infection treatment in patients with COPD and other vulnerable patients at risk of severe SARS-CoV-2 infection by applying the most recent evidence.

## Figures and Tables

**Table 1:** Baseline characteristics of patients hospitalized for SARS-CoV-2 infection between December 2021 and February 2024.

Characteristics		Before IPTW			After IPTW ^a^		
		CCS Only n = 15,299	RDV + CCS n = 24,084	SMD	CCS Only	RDV + CCS	SMD
Age group, years	18–49	313 (2.0%)	511 (2.1%)	0.0253	2.1%	2.1%	0
	50–64	2957 (19.3%)	4877 (20.2%)		19.9%	19.9%	
	≥65	12,029 (78.6%)	18,696 (77.6%)		78%	78%	
Sex	Female	8322 (54.4%)	13,087 (54.3%)	0.00114	54.4%	54.4%	−0.00033
	Male	6977 (45.6%)	10,997 (45.7%)		45.6%	45.6%	
Race	White	12,415 (81.1%)	19,750 (82.0%)	0.03038	81.7%	81.7%	0
	Black	2035 (13.3%)	2788 (11.6%)		12.2%	12.2%	
	Asian	117 (0.8%)	277 (1.2%)		1.0%	1.0%	
	Other	732 (4.8%)	1269 (5.3%)		5.1%	5.1%	
Ethnicity	Hispanic	667 (4.4%)	1400 (5.8%)	0.11731	5.3%	5.2%	0
	Non-Hispanic	13,515 (88.3%)	21,232 (88.2%)		88.3%	88.2%	
	Unknown	1117 (7.3%)	1452 (6.0%)		6.5%	6.5%	
Primary payor	Commercial	1018 (6.7%)	1941 (8.1%)	0.03818	7.5%	7.5%	0
	Medicare	12,426 (81.2%)	19,203 (79.7%)		80.2%	80.3%	
	Medicaid	1180 (7.7%)	1943 (8.1%)		7.9%	7.9%	
	Other	675 (4.4%)	997 (4.1%)		4.3%	4.3%	
Admission source	Transfer from SNF or ICF	573 (3.7%)	1028 (4.3%)	0.02667	4.1%	4.1%	−0.00273
	All other categories	14,726 (96.3%)	23,056 (95.7%)		95.9%	95.9%	
Bed size	<100	1325 (8.7%)	2078 (8.6%)	0.1098	8.7%	8.7%	0.02994
	100–199	2660 (17.4%)	4198 (17.4%)		17.4%	17.4%	
	200–299	3262 (21.3%)	4851 (20.1%)		20.5%	20.5%	
	300–399	3028 (19.8%)	4132 (17.2%)		18.1%	18.1%	
	400–499	1708 (11.2%)	2546 (10.6%)		10.8%	10.8%	
	500+	3316 (21.7%)	6279 (26.1%)		24.4%	24.4%	
Hospital location	Urban	12,812 (83.7%)	20,730 (86.1%)	0.06512	85.0%	85.1%	0.00221
	Rural	2487 (16.3%)	3354 (13.9%)		15.0%	14.9%	
Teaching hospital		5732 (37.5%)	9706 (40.3%)	0.05816	39.2%	39.2%	−0.00028
Region	Midwest	3984 (26.0%)	5953 (24.7%)	0.17766	25.2%	25.2%	0
	Northeast	1537 (10.0%)	3707 (15.4%)		13.4%	13.3%	
	South	8344 (54.5%)	11,864 (49.3%)		51.2%	51.3%	
	West	1434 (9.4%)	2560 (10.6%)		10.2%	10.2%	
Key co- morbidities	Obesity	4565 (29.8%)	7061 (29.3%)	−0.0114	29.5%	29.6%	0.00087
	COPD	15,299 (100.0%)	24,084 (100.0%)	0	100%	100%	0
	Cardiovascular disease	14,332 (93.7%)	22,193 (92.1%)	−0.05969	92.8%	92.7%	−0.00136
	Diabetes	6257 (40.9%)	9191 (38.2%)	−0.05598	39.2%	39.2%	−0.00014
	Renal disease	5515 (36.0%)	6415 (26.6%)	−0.20395	30.3%	30.3%	−0.00027
	Immunocompromised condition	2985 (19.5%)	4753 (19.7%)	0.00564	19.8%	19.7%	−0.00319
	Cancer	1224 (8.0%)	1989 (8.3%)	0.00944	8.2%	8.2%	−0.00025
	Lung cancer	452 (3.0%)	791 (3.3%)	0.01898	3.1%	3.2%	0.00813
ICU stay/General ward at baseline	Non-ICU	12,566 (82.1%)	19,559 (81.2%)	0.0239	81.6%	81.6%	0.00137
	ICU	2733 (17.9%)	4525 (18.8%)		18.4%	18.4%	
Admit diagnosis	Sepsis	72 (0.5%)	68 (0.3%)	−0.03075	0.4%	0.4%	0.00077
	Pneumonia	1307 (8.5%)	1831 (7.6%)	−0.03453	7.9%	7.9%	0.00116
Other treatments at baseline	Anticoagulants	11,175 (73.0%)	18,859 (78.3%)	0.12285	76.4%	76.3%	−0.00101
	Convalescent plasma	2 (0.0%)	16 (0.1%)	0.02677	0%	0%	0.00058
	CCS other than DEX	6982 (45.6%)	9501 (39.4%)	−0.1254	41.9%	41.9%	−0.00013
Baseline supplemental oxygen requirements	NSOc	5209 (34.0%)	7717 (32.0%)	0.09746	32.8%	32.8%	0
	LFO	5887 (38.5%)	9336 (38.8%)		38.6%	38.7%	
	HFO/NIV	3533 (23.1%)	6359 (26.4%)		25.2%	25.1%	
	IMV/ECMO	670 (4.4%)	672 (2.8%)		3.4%	3.4%	
CCI	Mean [SD]	3.9 [2.5]	3.5 [2.4]	−0.15648	3.7 [3.9]	3.6 [3.1]	−0.01587
	Median [IQR]	3.0 [2.0; 6.0]	3.0 [2.0; 5.0]		2.0 [2.0; 5.0]	2.0 [2.0; 5.0]	
	Range [LL, UL]	[1.0; 20.0]	[1.0; 20.0]		[1.0; 20.0]	[1.0; 20.0]	
CCS continued throughout the hospitalization without addition of antiviral, n (%)		13,664 (89.3%)	N.A.		89.0%	N.A.	
CCS among NSOc patients continued through the hospitalization without antiviral treatment, n (%)		4662 (89.5%)	N.A.		89.4%	N.A.	

Data are presented as n (%) before IPTW and as % after IPTW, unless otherwise indicated. CCI, Charlson comorbidity index; COPD, chronic obstructive pulmonary disorder; CCS, corticosteroid; DEX, dexamethasone; ECMO, extracorporeal membrane oxygenation; HFO, high-flow oxygen; ICU, intensive care unit; IMV, invasive mechanical ventilation; IPTW, inverse probability treatment weighting; IQR, interquartile range; LFO, low-flow oxygen; LL, lower limit; N.A., not applicable; NIV, non-invasive ventilation; NSOc, no supplementary oxygen charges; RDV, remdesivir; SNF, skilled nursing facility; SD, standard deviation; SMD, standardized mean difference; UL, upper limit. ^a^ After removing extreme propensity scores <0.05 and >0.95.

## Data Availability

The dataset for this study is from Premier, Inc. (https://www.premierinc.com/). Restrictions apply to the availability of these data, which were used under license for this study.

## Supplementary Materials

The following supporting information can be downloaded at: https://www.mdpi.com/article/10.3390/v17111438/s1, Table S1: Definitions of key study variables.; Table S2: Sensitivity analysis excluding those aged 18-49 years.
