# Supplementary material for: Clinical Outcomes of COPD Patients Hospitalized for SARS-Cov-2 Infection During the Omicron Era: Comparative Effectiveness of Initiating Remdesivir in Addition to Corticosteroids Versus Corticosteroids Alone
Source: Viruses. 2025 Oct 29;17(11):1438. doi: 10.3390/v17111438 (PMC12656847; doi:10.3390/v17111438)
Supplement: Supplementary file 1 [file viruses-17-01438-s001.zip › viruses-3890145-supplementary.pdf]

Viruses Supplementary Material

Clinical Outcomes of COPD Patients Hospitalized For SARS-Cov-2 Infection During The Omicron Era: Comparative Effectiveness Of Initiating Remdesivir in Addition to Corticosteroids versus Corticosteroids Alone

Neera Ahuja[1,*], Heng Jiang[2], Marc Milano[3], Roman Casciano[4], Ananth Kadambi[5], Thomas Oppelt[6], Fariborz Rezai[7], Martin Kolditz[8] and Veronika Müller[9] and Essy Mozaffari[10]

[1] Department of Internal Medicine, Stanford University School of Medicine, Palo Alto, California, USA

[2] Evidence and Access, Certara, Neuilly-sur-Seine, Île-de-France, France

[3] Newark Beth Israel Medical Center; Robert Wood Johnson Barnabas Health Care System, Somerset, Somerville, New Jersey, USA

[4] Evidence and Access, Certara, New York City, New York, USA

[5] Evidence and Access, Certara, Radnor, Pennsylvania, USA

[6] Gilead Sciences Inc., Foster City, California, USA

[7] Rutgers Medical School; System Director, Critical Care Medicine, RWJ Barnabas Health, New Jersey, USA

[8] Medical Department I, University Hospital Carl Gustav Carus of TU Dresden, Dresden, Saxony, Germany

[9] Department of Pulmonology, Semmelweis University, Budapest, Hungary

[10] Medical Affairs, Gilead Sciences, Foster City, California, USA

Supplementary Material

Table S1. Definitions of key study variables.

Key Study Variables		Definitions
**Remdesivir treatment**		Billing charges for treatment at baseline: Remdesivir;<br>ICD-10 procedure codes: XW033E5, XW043E5
**Key Comorbidities**	Obesity	ICD-10-CM diagnosis codes: E66, Z6825-Z6845
	COPD	ICD-10-CM diagnosis codes: J43, J44
	Cardiovascular disease (including hypertension)	ICD-10-CM diagnosis codes: I00-I99
	Diabetes	ICD-10-CM diagnosis codes: E10-E14
	Renal disease	ICD-10-CM diagnosis codes: I120, I131, N032, N033, N034, N035, N036, N037, N052, N053, N054, N055, N056, N057, N18, N19, N250, Z490, Z491, Z492, Z940, Z992
	Cancer	ICD-10-CM diagnosis codes: C00-C96
	Immunocompromised condition	ICD-10-CM code for cancer (C00-C96), transplant (Z94.x), hematologic malignancies (C81.x, C82.x, C83.x, C84.x, C85.x, C88.x, C90.x, C91.x, C92.x, C93.x, C94.x, C95.x, C96.x) , primary immunodeficiencies (D80.x, D81.x, D82.x, D83.x, D84.x, G11.3, E70.330, D71.x, D70.x), asplenia (Q89.01, Z90.81), toxic effects of antineoplastics (T45.1x), bone marrow failure/aplastic anemia (D61.x), severe combined immunodeficiencies (D80.x, D81.x, D82.x, D83.x, D84.x, D86.x, D89.0, D89.1, D89.2, D89.3 , D89.4x, D89.81, D89.82, D89.89, D89.9), HIV (B20), patients with chronic graft-versus-host disease or who are taking immunosuppressive medications for another indication (Z89.8x, Z79.52, Z79.61, Z79.62x, Z79.63x, Z79.64, Z79.69, Z79.810, Z79.811, Z79.818)
**Supplemental oxygen requirements**	IMV/ECMO	Billing charges for devices: invasive mechanical ventilation, tracheostomy, endotracheal tube, intubation, extracorporeal membrane oxygenation
	HFO/NIV	Billing charges for devices: negative-pressure ventilation, positive-pressure ventilation, CPAP, BiPAP, high flow system via nasal cannula, venturi face mask, rebreather, non-rebreather mask, positive expiratory pressure
	LFO	Billing charges for devices/oxygen supply: Simple face mask, oxygen pendant, low flow system via nasal cannula, oxygen supply. NSOc patients at baseline who are admitted to the ICU within the first two days are reclassified into the LFO group.
	NSO	No billing charges for IMV/ECMO, HFO/NIV, or LFO at baseline.

**Admitting Diagnosis**	Sepsis	ICD-10-CM diagnosis codes: A021, A327, A40, A41, A427, A5486, B377, R6520, R6521, T8144X,
	Airway infection or pneumonia	ICD-10-CM diagnosis codes: J13, J14, J80, J110, J120, J121, J122, J123, J129, J151, J154, J156, J157, J158, J159, J168, J180, J181, J182, J188, J189, J9600, J9601, J9690, J9691, J9620, J9621, J1281, J1282, J1289, J1529, J15211 And ICD-10 diagnosis description exactly matching "HYPOXEMIA"
**Other treatments**	Anticoagulants	Billing charges for treatment: Treatments used at baseline: apixaban, argatroban, desirudin, lepirudin, dabigatran, danaparoid, edoxaban, tinzaparin, heparin (excluding use of heparin flush), ardeparin, bivalirudin
	Corticosteroids	Billing charges for treatment: prednisone, prednisolone, methylprednisolone, hydrocortisone, dexamethasone,
	Convalescent plasma	Billing charges for treatment at baseline: convalescent plasma; ICD-10 procedure codes: XW13325, XW14325
	Baricitinib	Billing charges for treatment: Baricitinib; ICD-10 procedure codes: XW0DXM6, XW0H7M6, XW0G7M6
	Tocilizumab	Billing charges for treatment: Tocilizumab; ICD-10 procedure codes: XW033H5, XW043H5
	Oral antivirals	Billing charges for treatment: nirmatrelvir ritonavir, molnupiravir

Table S2. 14- and 28-day mortality in patients with COPD hospitalized with SARS-CoV-2 infection initiated on both RDV + CCS or CCS alone upon admission (after IPTW, sensitivity analysis excluding those aged 18-49 years).

	**aHR [95% CI]**	**P-value**
14-day mortality	0.73 [0.67 - 0.80]	<0.0001
28-day mortality	0.76 [0.70 - 0.82]	<0.0001

Estimates were adjusted for age, admission month, hospital admission ward (documented bed charges for ICU/step-down unit versus general ward), and time-varying covariates for treatments initiated after the baseline period, such as baricitinib, tocilizumab, oral antivirals, or CCS other than DEX. aHR, adjusted hazard ratio; CCS, corticosteroids; CI, confidence interval; COPD, chronic obstructive pulmonary disease; DEX, dexamethasone; ICU, intensive care unit; IPTW, inverse probability of treatment weighting; RDV, remdesivir.
